# Post–Chikungunya Virus Infection Musculoskeletal Disorders: Syndromic Sequelae after an Outbreak

**DOI:** 10.3390/tropicalmed6020052

**Published:** 2021-04-15

**Authors:** Hisham A. Imad, Wasin Matsee, Sajikapon Kludkleeb, Punyisa Asawapaithulsert, Juthamas Phadungsombat, Emi E. Nakayama, Keita Suzuki, Pornsawan Leaungwutiwong, Watcharapong Piyaphanee, Weerapong Phumratanaprapin, Tatsuo Shioda

**Affiliations:** 1 Mahidol-Osaka Center for Infectious Diseases, Faculty of Tropical Medicine, Mahidol University, Bangkok 10400, Thailand; juthamasps@gmail.com (J.P.); emien@biken.osaka-u.ac.jp (E.E.N.); shioda@biken.osaka-u.ac.jp (T.S.); 2 Department of Viral Infections, Research Institute for Microbial Diseases, Osaka University, Osaka 565-0871, Japan; keita-s@ml.tanaka.co.jp; 3 Bangkok Hospital for Tropical Diseases, Faculty of Tropical Medicine, Mahidol University, Bangkok 10400, Thailand; wasin.mat@mahidol.edu (W.M.); sajikapon.klu@mahidol.ac.th (S.K.); punyisa.asa@mahidol.ac.th (P.A.); watcharapong.piy@mahidol.ac.th (W.P.); weerapong.phu@mahidol.ac.th (W.P.); 4 Department of Clinical Tropical Medicine, Faculty of Tropical Medicine, Mahidol University, Bangkok 10400, Thailand; 5 Point of Care Testing Products Business Unit, TANAKA Kikinzoku Kogyo, Hiratsuka 254-0076, Japan; 6 Tropical Medicine Diagnostic Reference Laboratory, Faculty of Tropical Medicine, Mahidol University, Bangkok 10400, Thailand; pornsawan.lea@mahidol.ac.th; 7 Department of Microbiology and Immunology, Faculty of Tropical Medicine, Mahidol University, Bangkok 10400, Thailand

**Keywords:** acute febrile illness, *Alphavirus*, chikungunya virus, post-chikungunya musculoskeletal disorder, post-chikungunya chronic inflammatory rheumatism

## Abstract

The Chikungunya virus is a re-emerging mosquito-borne alphavirus. Outbreaks are unpredictable and explosive in nature. Fever, arthralgia, and rash are common symptoms during the acute phase. Diagnostic tests are required to differentiate chikungunya virus from other co-circulating arboviruses, as symptoms can overlap, causing a dilemma for clinicians. Arthritis is observed during the sub-acute and chronic phases, which can flare up, resulting in increased morbidity that adversely affects the activities of daily living. During the 2019 chikungunya epidemic in Thailand, cases surged in Bangkok in the last quarter of the year. Here, we demonstrate the chronic sequelae of post-chikungunya arthritis in one of our patients one year after the initial infection. An inflammatory process involving edema, erythema, and tenderness to palpation of her fingers’ flexor surfaces was observed, with positive chikungunya IgG and negative IgM tests and antigen. The condition produced stiffness in the patient’s fingers and limited their range of motion, adversely affecting daily living activities. Resolution of symptoms was observed with a short course of an anti-inflammatory agent. More research is required to determine whether sanctuaries enable chikungunya virus to evade the host immune response and remain latent, flaring up months later and triggering an inflammatory response that causes post-chikungunya arthritis.

## 1. Introduction

Arboviral infections exhibit many etiologies and cause acute febrile illnesses in tropical and subtropical regions. These infections are considered “neglected tropical diseases” that place over one billion people worldwide at risk of illness [1]. The co-existence of these pathogens in endemic regions poses a conundrum in clinical practice [2]. Due to similarities in their presentation, these viruses can be easily misidentified [3]. The use of definite laboratory diagnostics, either molecular techniques or serology, is therefore crucial in narrowing down the differential diagnosis of acute febrile illnesses or, more precisely, acute undifferentiated febrile illnesses. Infections involving the majority of arboviral etiologic agents such as the dengue viruses, Zika, and chikungunya virus (CHIKV), frequently manifest as an abrupt-onset illness with non-localizing signs and symptoms; the infections are self-limited, and patients typically recuperate without the use of any particular therapeutics [3,4,5]. Conversely, of the three sympatric pathogens described, CHIKV causes more morbidity and arthritis during chronic infection. 

CHIKV is a mosquito-borne *Alphavirus* of the *Togaviridae* family, and it was first detected along the forest fringes of modern-day Tanzania [6]. Since its discovery, the virus has been more commonly known for its re-emergence and resurgence in tropical and subtropical regions. In addition, CHIKV is characterized by unpredictable outbreak patterns that are explosive in nature, short-lived, and sporadically limited during inter-epidemic periods [7]. The virus has three specific lineages, and polymorphisms that have arisen in the viral genome over the last decade have produced sub-lineages that are detected with increasing frequency across the globe [8]. The sylvatic transmission cycle has been well characterized, but details regarding the urban cycle and identity of the reservoir maintaining the virus remain unclear. However, the virus is amplified and sustained in humans during outbreaks. 

After an incubation period of 2–10 days, over 70% of patients become symptomatic with a febrile arthritogenic infection that resolves naturally, providing life-long immunity [9]. Fatalities from CHIKV infection are rare, but the virus can cause high morbidity. Arthralgia and arthritis can affect large joints and proximal joints and is polyarticular in nature, with symmetric or asymmetric polymorphic involvement. Arthralgia is the predominant feature, but atypical infections can involve other organs. Individuals of extreme age or with underlying comorbidities develop a more severe infection, leading to multi-system involvement. Common laboratory features include leukopenia and thrombocytopenia, with lymphopenia and neutrophilia varying with the degree of viremia.

In terms of therapeutic management of the arthritides caused by CHIKV, infections are classified as either acute, sub-acute, or chronic [10]. The acute phase lasts for three weeks, whereas the sub-acute phase begins at three weeks of illness and lasts to the end of three months, and protracted chronic infection is diagnosed when the illness persists beyond three months with symptomatic sequelae. This syndrome can be recurrent, with flare-ups during the sub-acute phase and chronic period. These flare-ups can involve episodic relapses and periods of remission of musculoskeletal symptoms reminiscent of rheumatism, causing debilitating and restricted ambulation that adversely impacts an affected individual’s quality of life. As observed previously, the median number of relapses is two, with a range of 1–20, and the median delay between relapses is four weeks (range of 1–99 weeks) [11]. Over 60% of individuals affected by the virus can develop chronicity [12]. Those most at risk of developing chronic infection include the elderly, women, and individuals with an underlying musculoskeletal disorder [13]. Moreover, increasing evidence suggests that severity and chronicity vary with the viral genotype [14].

Details of the mechanism leading to persistent and recurrent inflammatory arthritis post-CHIKV infection are poorly understood. Historically, many viruses have been described as causing arthritis or suspected of triggering an autoimmune response after infection [15]. The first description of a post-CHIKV rheumatic disorder was in South Africa after an outbreak in the 1970s [16]. The host responses in post-CHIKV inflammatory arthritis and rheumatoid arthritis involve the expression of the same pro-inflammatory cytokines and chemokines, with comparable clinical findings [17]. The majority of cases fail to meet the criteria for rheumatoid arthritis during articular inflammatory flare-ups, with seronegative status for rheumatism [18]. This makes it difficult to recognize the early onset of rheumatoid arthritis and initiate appropriate targeted treatment in a timely manner. However, cases of erosive rheumatoid arthritis were reported following a large Indian Ocean CHIKV epidemic [19]. 

Here, we report the chronic sequelae of post-CHIKV arthritis in a patient one year after the initial infection.

## 2. Materials and Methods 

The patient was a participant of one of our previous studies conducted at the Bangkok Hospital for Tropical Diseases during the CHIKV outbreak in 2019 [3]. After becoming symptomatic again in 2020, the patient returned to the Fever Clinic. The Mahidol-Osaka Center for Infectious Diseases at the Faculty of Tropical Medicine, Mahidol University, provided diagnostic support for the CHIKV infection. The patient’s medical chart was reviewed retrospectively to extract the clinical data, and the patient was closely followed up until resolution of her symptoms. We used a prototype lateral-flow immunochromatography rapid point-of-care test kit to detect the CHIKV envelope protein 1 (E1) antigen, which was subsequently confirmed via real-time reverse transcription polymerase chain (RT-PCR) analysis [20,21]. Other serologic tests included anti-CHIKV immunoglobulin M (IgM) and immunoglobulin G (IgG) (SD Biosensor, Inc. Gyeonggi-do, Korea), dengue non-structural protein 1 (NS1) antigen (Biosynex, Swiss S.A, Fribourg, Switzerland), and anti-DENV IgM and IgG (S, Bioline, Sankt Ingbert, Germany). To exclude other possible co-circulating arboviruses in Thailand, we also performed real-time RT-PCR for both dengue and Zika virus. 

## 3. Case Report

A case of post-CHIKV arthritis in a patient from our CHIKV patient cohort is presented. A Thai woman in her mid-forties presented to the Fever Clinic at Bangkok Hospital for Tropical Diseases following surges of CHIKV cases during the 2019 outbreak in Thailand. She had no underlying past medical history of diabetes, hypertension, chronic lung disease, chronic kidney disease, ischemic heart disease, or any known musculoskeletal disorder and was not on any regular treatment or consuming any supplements. She denied any recent travel history outside of Bangkok, coming into contact with animals (rodents), or exposure to floods, and she had not received any blood transfusion over the past four weeks. She presented on her third day of illness with complaints of an abrupt onset of high-grade fever (39 °C) associated with chills, arthralgia, myalgia, rash, finger stiffness, and difficulty walking. On examination, she was conscious and coherent with time, place, and person. There was no noticeable pallor or jaundice and no evidence of dehydration or any palpable lymphadenopathy, but injection of the conjunctiva, erythema over the cheeks, and erysipelas of the pinnae were noted (Figure S1). 

Physical examination of the cardiovascular system was normal except for tachycardia. Examination of the respiratory and central nervous systems was unremarkable. The patient’s abdomen was soft, with no evidence of hepato-splenomegaly. A maculopapular rash was observed predominantly over the trunk, which was described as pruritic and suggestive of a centrifugal distribution. The arthralgia involved large joints (knees), without swelling or joint effusion as well as peripheral joints (wrists and phalanges). There was swelling of the proximal phalangeal and metacarpophalangeal joints, with restricted range of motion. A pain severity score of 8/10 was reported. The myalgia was reported as generalized, not predominantly affecting the lower or upper extremities. Laboratory investigation results at the time of presentation are shown in Table 1.

CHIKV infection was suspected, and the patient was screened using a novel antigen test that detects the E1 protein; CHIKV infection was later confirmed via real-time RT-PCR analysis. With possible co-infections ruled out and without prior known allergies to non-steroidal anti-inflammatory drugs (NSAIDs), naproxen was prescribed at a daily dose of 1000 mg, along with an antihistamine (hydroxyzine 25 mg), for three days, to which she responded well, reporting much relief at the follow-up consultation on the fifth day of illness. Complete resolution of symptoms was noted on follow-up nine days after onset. 

**Figure 1:**
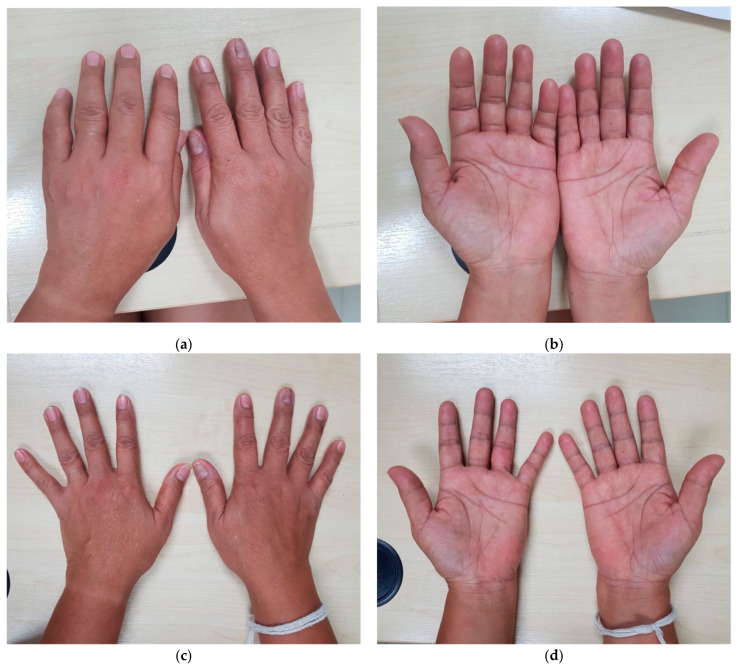
Images showing fingernail discoloration and post-CHIKV arthritis of the proximal interphalangeal joints and resolution of arthritis on the follow-up. (**a**,**b**) Flexor tenosynovitis, post-CHIKV musculoskeletal disorders (MCD) (6 October 2020); (**c**,**d**) resolution of tenosynovitis after treatment with a NSAID (8 October 2020).

After almost a year without further flare-ups of symptoms characteristic of CHIKV, the patient returned to the Fever Clinic with new onset of febrile illness. She presented on the second day of illness with complaints of fever, chills, arthralgia, and unilateral swelling and stiffness of her left hand. On examination of the affected limb, there was swelling of the proximal interphalangeal joints extending to the metacarpophalangeal joints, with a limited range of motion (Figure 1). 

**Figure 2:**
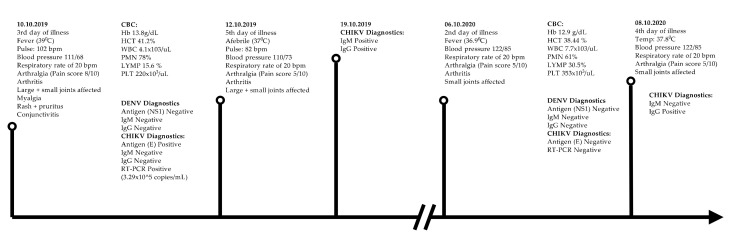
Elapsed time between the initial presentation to the hospital and relapse of symptoms, clinical findings, and investigations performed during each visit.

After a brief interview about possible exposure, travel history, and trauma to the left hand, routine investigations were conducted. The elapsed time between the initial presentation to the hospital and the relapse of symptoms including clinical manifestations and investigations performed during each visit are depicted in Figure 2.

## 4. Discussion

We describe a case involving typical musculoskeletal manifestations of acute CHIKV infection including cutaneous manifestations. The Milians ear sign is increasingly recognized during the acute phase of CHIKV infection [22]. Nevertheless, non-specific symptoms such as non-purulent conjunctivitis can resemble those associated with other endemic virus infections [5]. Clinicians working in fever clinics or travel clinics caring for patients residing in endemic regions or travelers returning from tropical regions should carefully consider infection with all possible tropical pathogens that could present as an acute undifferentiated febrile illness. It is essential to inquire about possible exposures, which can help narrow the differential diagnosis of tropical viruses or bacteria such as *Leptospira* or *Rickettsia*, for which effective treatments exist. For most arboviruses, rapid point-of-care diagnostics are helpful for prompt identification of suspected cases of dengue, CHIKV, or Zika.

As alluded to in the Introduction, details of the mechanism underlying the chronicity and flare-up of symptoms with CHIKV are unclear. However, it has been demonstrated that factors such as viremia, advanced age at the onset of illness, female gender, comorbidities, pre-existing rheumatism or arthropathy, and genetic predisposition may contribute to the persistence of symptoms in CHIKV infection [23]. Furthermore, the East Central South African (ECSA) lineage and Asian lineage reportedly cause long-lasting musculoskeletal disorders [24]. Similarly, post-CHIKV arthritis can occur during infection with the ECSA Indian Ocean sub-lineage, as observed in the present case [3]. Inflammatory polyarthritis is the most common long-term sequelae to occur with chikungunya infection [12,25,26]. However, there are reports of alopecia, skin hyper-pigmentation, chronic fatigue, and depression to occur as persisting sequelae [27,28,29]. Other long-term sequelae after a viral insult to the central nervous system by the CHIKV include persistent neurological sequelae manifesting as epilepsy or post-infectious dementia. Encephalitis or encephalopathy in neonates and children have a worsened neurocognitive function with severe development deficits [30]. Albeit in small numbers, ocular complications leading to loss of visual acuity and permanent neurological disability after acute disseminated encephalomyelitis have previously been reported [31].

Unlike animal models in which pathologies and persistence of viral RNA have been demonstrated in joint tissue in vivo during acute CHIKV infection, no viable viruses or viral genetic material have been found within the joint articular spaces in humans, except in a limited number of cases in which the CHIKV antigen was identified in perivascular macrophages [32,33]. This lack of detection might be due a robust innate type 1 immune response that directs macrophages to clear up the viruses or to a decreased threshold of detection for extremely low viremia levels [34]. Interferons are known for their antiviral properties; any interferon deficiencies observed with increasing age could contribute to the severity of the infection [35]. In addition, interferons function poorly at lower temperatures, which promotes arthritis in infections with other alphaviruses [35]. Similarly, interferon dysfunction could play a role in the peripheral articular joints predominantly involved during CHIKV infection. As described previously in patients with CHIKV, levels of circulating anti-inflammatory cytokines and cytotoxic T-cell activity are low during post-CHIKV arthritis [33]. Individuals with a compromised immune system due to diabetes or immunosuppressive therapy are vulnerable to development of chronic sequelae of inflammatory arthritis post-CHIKV infection.

Although IgM can be detected as early as the fourth day after the onset of symptoms and aids in viral clearance, IgM persistence is also reportedly associated with destructive arthropathies during CHIKV infection [36]. In general, the IgM level decreases to below the detection limit within three to four months, and IgG persistence ensures life-long immunity. Some researchers have described a slightly extended IgM depletion trend lasting until the end of 18 months [37]. In our case, IgM and IgG were detected during the second week of the acute phase, but IgM became undetectable by the end of 12 months, but IgG was positive, facilitating immunologic memory. After an acute CHIKV infection, the common observation is the resolution of symptoms during the acute phase, but protraction of symptoms into the chronic phase with relapse can occur within three months. In contrast, in our case, although the patient’s symptoms resolved after the acute CHIKV infection, after approximately one year, the patient presented again with symptoms consistent with post-CHIKV rheumatic and musculoskeletal disorders (pCHIKV-RMSD). 

An inflammatory process involving edema, erythema, and tenderness to palpation of the flexor surfaces of her fingers was noted. This inflammatory response produced stiffness in her fingers and limited their range of motion, thus adversely affecting her daily living activities. The term pCHIKV-RMSD was coined by rheumatologists. For specific targeted therapeutic management, pCHIKV-RMSD was partitioned into two additional categories. These include post-CHIKV musculoskeletal disorders (pCHIKV-MSD), which respond to anti-inflammatory agents, and post-CHIKV de novo chronic inflammatory rheumatism (pCHIKV-CIR), which is characterized by the presence of rheumatism without evidence of articular disorders prior to CHIKV infection [38].

Limitations in this case report include the unavailability of inflammatory biomarker data (rheumatoid factor, anti–citrullinated protein antibodies, antinuclear antibodies, C-reactive protein, erythrocyte sedimentation rate, uric acid levels, or human leukocyte antigen antibodies) and imaging (x-rays or magnetic resonance imaging of the articular joints) to determine the presence of any degenerative or erosive arthritis. It is important to consider other *Alphaviruses* that can exhibit persistent arthritides after an acute infection. The Mayaro virus and Ross River virus (RRV) are still geographically restricted to South America and Australia including the Southwestern Pacific islands [39]. The Barmah Forest virus is found only in the Australian mainland, and outbreaks of the O’ nyong-nyong virus have occurred in East and West Africa [40]. As the patient declared no recent travel history, we did not consider these *Alphaviruses* capable of manifesting as arthritides. The Sindbis virus group is widely distributed in Africa, Asia, and Australia, with an increased endemicity in Northern Europe. The clinical presentation was not suggestive of other cosmopolitan viruses such as rubella, cytomegalovirus, or hepatitis. More research is thus required to determine whether there are sanctuaries for CHIKV in which the virus can evade the host immune response and remain latent, flaring up months later and triggering inflammatory responses leading to pCHIKV-MSD or pCHIKV-CIR. The new onset of inflammation in the patient’s fingers was consistent with pCHIKV-MSD, with rapid response to a short course of NSAIDs. Treatment strategies should take a combined approach involving the primary care physician and a rheumatologist to optimize the management of such cases. 

## Figures and Tables

**Table 1:** Clinical and laboratory data during acute infection (2019) and upon flare-up of symptoms (2020).

Year of Presentation	2019		2020
Day of illness	3rd day	5th day	2nd day
History of fever	Yes	No	Yes
Temperature °C	39	37	36.9
Pulse, beats per minute	102	110	122
Systolic blood pressure, mmHg	111	110	122
Diastolic blood pressure, mmHg	68	73	85
Arthralgia	Yes	Yes	Yes
Arthralgia pain score, (0/10)	8/10	5/10	5/10
Arthritis	Yes	No	Yes
Myalgia	Yes	No	No
Fatigue	Yes	No	No
Rash	Yes	No	No
Pruritus	Yes	No	No
Headache	Yes	No	Yes
Diarrhea	No	No	No
Vomiting	No	No	No
Nausea	No	No	No
Conjunctivitis	Yes	No	No
Dengue NS1 antigen	Negative		Negative
Dengue IgM antibody	Negative		Negative
Dengue IgG antibody	Negative		Negative
Chikungunya E1 antigen	Positive		Negative
Chikungunya IgM	Negative	Positive	Negative
Chikungunya IgG	Negative	Positive	Positive
CHIKV real time RT-PCR (Ct value)	19.43		Negative
CHIKV viral load, copies/mL	3.29 × 10^5^		Not detected
Hemoglobin g/dL	13.8		12.9
Hematocrit %	41.2		38.3
Leukocytes/µL	4100		7700
Neutrophils/µL	3218		4750
Lymphocytes/µL	639		2348
Monocytes/µL	231		250
Eosinophils/µL	54		53
Basophils/µL	54		16
Platelets/µL	220,000		353,000

NS1 = non-structural protein 1; IgM = immunoglobulin M; IgG = immunoglobulin G; E1 = envelope protein 1; Ct = cyclic threshold.

## Data Availability

The data presented in this study are available on request from the corresponding author. The data are not publicly available to ensure the privacy of the study participant.

## Supplementary Materials

The following are available online at https://www.mdpi.com/article/10.3390/tropicalmed6020052/s1, Figure S1. Images taken at presentation to the Fever Clinic during the acute CHIKV infection (October 2019). (**a**) Non-purulent conjunctivitis of both eyes and erythema over the cheeks, (**b**) erythema of the pinna (Milians ear sign), (**c**) bilateral swelling of metacarpophalangeal joints and ambulation in a wheel chair due to debilitating arthralgia of the knees.
