# Supplementary material for: Post–Chikungunya Virus Infection Musculoskeletal Disorders: Syndromic Sequelae after an Outbreak
Source: Trop Med Infect Dis. 2021 Apr 15;6(2):52. doi: 10.3390/tropicalmed6020052 (PMC8167736; doi:10.3390/tropicalmed6020052)
Supplement: Supplementary file 1 [file tropicalmed-06-00052-s001.zip › tropicalmed-1178923-supplementary.pdf]

Tropical Medicine and Infectious Disease

MDPI

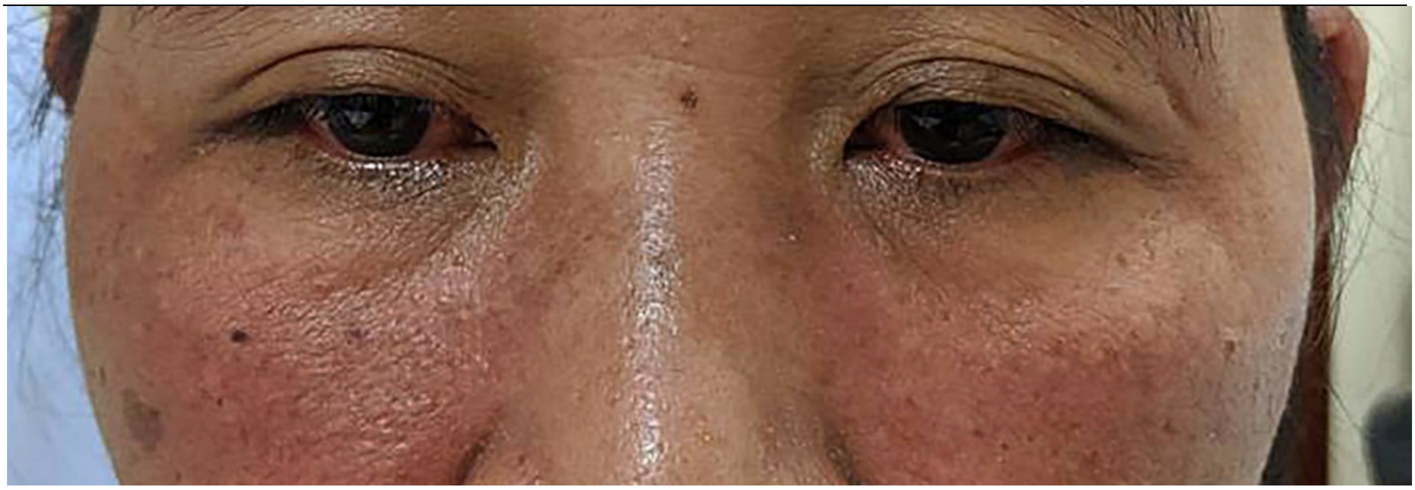

(**a**)

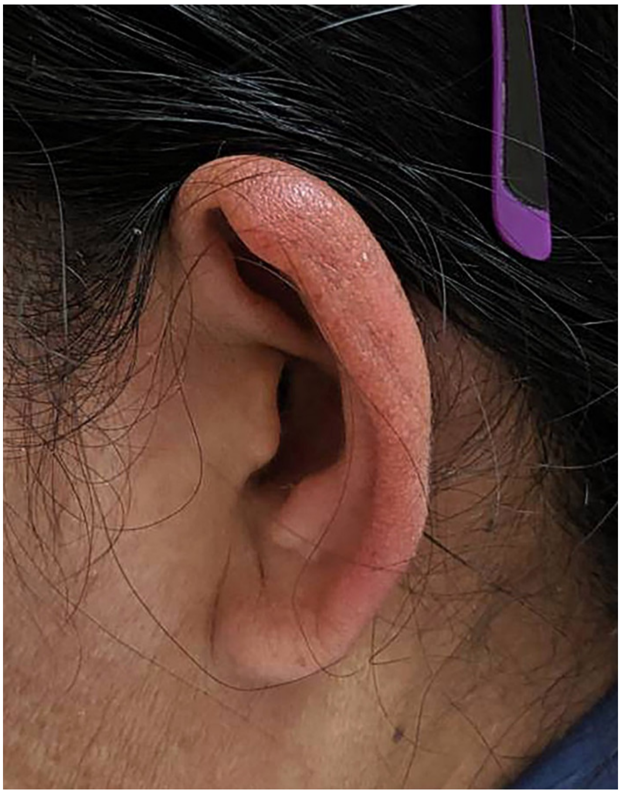

(**b**)

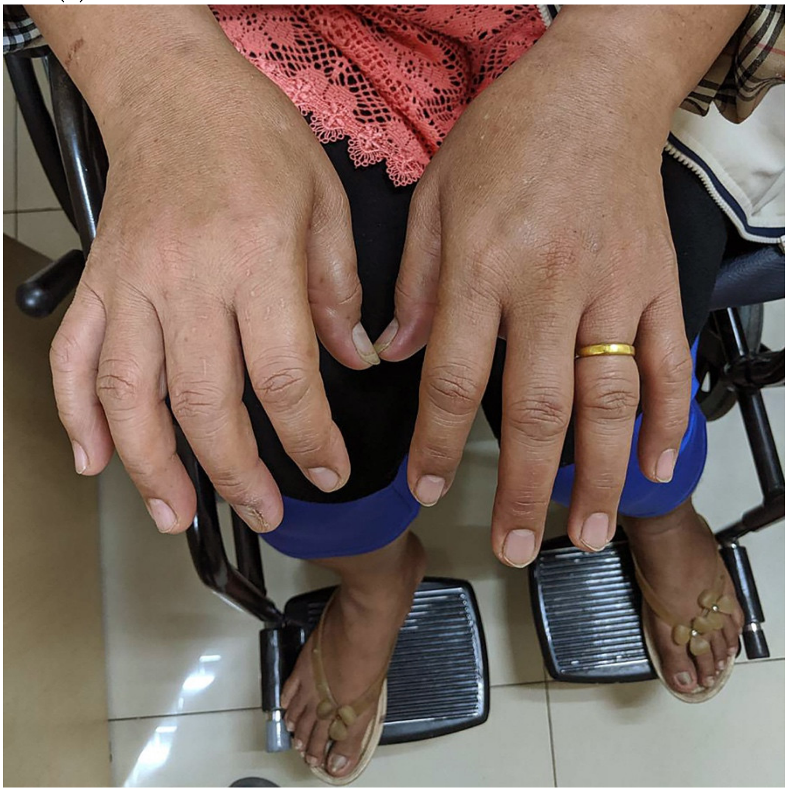

(**c**)

Figure S1 Images taken at presentation to the Fever Clinic during the acute CHIKV infection (October 2019). (**a**) Non-purulent conjunctivitis of both eyes and erythema over the cheeks, (**b**) erythema of the pinna (Milians ear sign), (**c**) bilateral swelling of metacarpophalangeal joints and ambulation in a wheel chair due to debilitating arthralgia of the knees
